# Asthma and Obesity in Children

**DOI:** 10.3390/biomedicines8070231

**Published:** 2020-07-21

**Authors:** Francesco Sansone, Marina Attanasi, Sabrina Di Pillo, Francesco Chiarelli

**Affiliations:** 1 Department of Pediatrics, Pediatric Allergy and Pulmonology Unit, University of Chieti-Pescara, 66100 Chieti, Italy; francesco.sansone001@alumni.unich.it (F.S.); marina.attanasi@unich.it (M.A.); sabrinadipillo@gmail.com (S.D.P.); 2 Pediatric Research Unit, University of Chieti-Pescara, 66100 Chieti, Italy

**Keywords:** obesity, asthma, lung function, insulin resistance, atopy, dyslipidemia

## Abstract

Asthma and obesity are two major chronic diseases in children and adolescents. Recent scientific evidence points out a causative role of obesity in asthma predisposition. However, studies assessing the real impact of excessive weight gain on lung function in children have shown heterogeneous results. In this review, the pathological mechanisms linking obesity and development of asthma in children are summarized and factors influencing this relationship are evaluated. Common disease modifying factors including age, sex, ethnicity, development of atopic conditions, and metabolic alterations significantly affect the onset and phenotypic characteristics of asthma. Given this, the impact of these several factors on the obesity–asthma link were considered, and from revision of the literature we suggest the possibility to define three main clinical subtypes on the basis of epidemiological data and physiological–molecular pathways: obese-asthmatic and atopy, obese-asthmatic and insulin-resistance, and obese-asthmatic and dyslipidemia. The hypothesis of the different clinical subtypes characterizing a unique phenotype might have an important impact for both future clinical management and research priorities. This might imply the necessity to study the obese asthmatic child with a “multidisciplinary approach”, evaluating the endocrinological and pneumological aspects simultaneously. This different approach might also make it possible to intervene earlier in a specific manner, possibly with a personalized and tailored treatment. Surely this hypothesis needs longitudinal and well-conducted future studies to be validated.

## 1. Introduction

Nowadays, asthma and obesity represent two of the major chronic diseases in children and adolescents. The International Study of Asthma and Allergies in Childhood, an international multicenter epidemiological study, showed that asthma prevalence worldwide is slightly increasing, even if there are striking differences among countries [1]. Current wheezing (defined as at least one episode of wheezing in the last 12 months) has become a major health issue in Latin American countries, Western Europe, Tunisia, Morocco, and Algeria, whereas English-speaking countries have experienced a slight reduction in prevalence trend throughout the last decade [1]. Similarly, obesity in children of all ages has increased over the last four decades, with an eightfold increase in 5–19 year old individuals between 1975 and 2016 and a twofold increase in younger children between 1980 and 2015 [2]. There is relevant heterogeneity in prevalence trends among different geographic areas, with a flattening in the last decade among countries with high socio-economic status and a steeper rise in Africa, Asia, and Latin America [2]. Traditionally, asthma in children has been associated with atopy and bronchial eosinophilic inflammation. Recent studies have shown that despite new treatment options in asthma management (including biologic drugs and new inhalant combination-strategies) there are still 5–10% of patients who present with a difficult-to-treat phenotype, characterized by more exacerbations and bronchial hyper-reactivity that is non-respondent to standard therapies [3]. The parallel increase in the prevalence of pediatric obesity through the last decade has raised concerns about a possible correlation between obesity and asthma in children and adolescents, heading to an intensive study of the topic by many research groups all over the world.

The deeper we explore the link between asthma and obesity, the more it appears to be complex and tangled, involving many different factors such as insulin resistance, dyslipidemia, fat distribution, and dietary habits. For example, insulin resistance has been found to affect respiratory function independently from body mass index (BMI) and other obesity-related parameters [4,5].

The aim of this review is to critically summarize the current evidence on the obese asthmatic phenotype in children, allowing us to describe three main molecular pathophysiological mechanisms that might hypothetically underpin different clinical subtypes.

## 2. Materials and Methods

We searched for articles on PubMed using the keywords “obesity”, “asthma”, “lung function”, “insulin resistance”, “dyslipidemia”, and “children”. In particular, we used the following search terms and logic: “*obesity AND asthma OR lung function AND insulin resistance OR hyperinsulinemia AND dyslipidemia AND children OR childhood OR pediatric age OR infancy*”. Further studies were obtained through the references of some papers. Articles were selected according to their title and abstract, using eligibility criteria. The inclusion criteria were being in the English language; pediatric study population (age range 0–18 years old); type of study: narrative and systematic reviews, longitudinal retrospective and prospective studies, and randomized control trials. Adult studies, case reports, expert opinions, and manuscripts published in a language other than English were excluded. We selected 76 papers from a total of 99 inherent articles. The final reference list was developed on the basis of originality and relevance to the broader scope of this review.

## 3. Results and Discussion

### 3.1. Asthma Endotypes

Nowadays, asthma is an umbrella term encompassing different pathological manifestations that share variable degrees of bronchial inflammation, hyperreactivity, and remodeling [6]. Indeed, there is great heterogeneity in clinical manifestations, and even molecular pathways implicated in asthma pathogenesis are different among affected subjects. On the basis of molecular patterns, it is possible to describe two major endotypes of asthma: the T2-high or type 2 endotype, and the non-T2-high or T2-low endotype.

The T2-high endotype represents the classic form of asthma, characterized by eosinophilic airway infiltration and inflammation. The inflammatory cascade is activated by airway epithelial cells (AECs), which express a number of cytokines, named alarmins, after allergen exposure. Alarmins include thymic stromal lymphopoietin (TSLP), interleukin (IL)-25, and IL-33, whose main function is the activation of innate lymphoid cells type 2 (ILC2s) and antigen-presenting cells (APCs) [7]. ILC2s express high levels of IL-5 and IL-13 (responsible for Th2 response) and are implicated in airway remodeling [8]. APCs present the allergen fragments to cluster of differentiation(CD)4+ lymphocytes, inducing a Th2 polarization with production of IL-4, IL-5, and IL-13. These cytokines, in turn, determine eosinophil migration into the site of inflammation, with consequent release of cytotoxic proteins (major basic protein, eosinophil peroxidase, eosinophil cationic protein, eosinophil-derived neurotoxin), bronchoconstrictors (cysteinyl leukotrienes), and remodeling activating factors [9,10].

The T2-low endotype is characterized by the absence of eosinophilic infiltrates, principal biomarkers of Th2 response [6]. Affected individuals may show a neutrophilic sputum sample or a paucigranulocytic one (normal eosinophils and neutrophils count). The inflammatory response shows a Th1 polarization with the expression of high levels of interferon (IFN)-γ. A Th17 immune response may be observed in a subgroup of patients with high levels of IL-17, causing neutrophilic infiltration, airway hyperreactivity (AHR) and steroid resistance. Asthmatic patients with T2-low endotype are frequently steroid-resistant and have more exacerbations or even fixed airflow obstruction.

Traditionally, the T2-high endotype was associated with early-onset and atopic asthma, typical of childhood and adolescence. The T2-low endotype characterized the late-onset and non-atopic phenotype, typical of adults who present many comorbidities, such as smoking and obesity. Table 1 offers a schematic overview of the most important cytokines involved in T2-high and T2-low immune responses.

### 3.2. Obesity and Asthma

Two meta-analyses, published in 2013 and 2019, focused on the obese asthmatic phenotype and assessed the positive correlation between asthma and childhood obesity or overweight [12,13]. The first one included only six prospective studies and found a 20% increase in asthma incidence in overweight children and a twofold increase risk in obese children [12]. This correlation was stronger in boys until puberty. The second meta-analysis, which included 18 prospective studies, calculated a 20% risk increment in overweight children and a 40% risk increment for obese children [13]. There was a noticeably clear difference in gender data between the two meta-analyses, with the most recent one pointing out a stronger correlation among girls [13]. Deng et al. ascribes this contrasting finding to the study population heterogeneity, characterized by a very variable age span [13]. Furthermore, it is important to mention other potential sources of heterogeneity, such as the different subgroup analyses (e.g., according to weight category), sample size, ethnicity, region, diagnosis of asthma, follow-up point, and source of controls. To better understand this particular asthmatic phenotype in children, it is necessary to carry out further longitudinal large and well-conducted studies with a more homogenous population and a fewer number of different confounders.

BMI is the most common adopted parameter to evaluate weight range in both adults and children since it is easy to calculate. In children, overweight is defined as a BMI above the 85th percentile and obesity as a BMI above the 95th percentile [14]. Unfortunately, BMI does not distinguish fat mass from lean mass or subcutaneous from visceral adiposity [15], although fat mass distribution is more strongly associated with adverse health risks [16]. Studies that assessed the obesity–asthma link using more detailed measurements of total and abdominal fat mass distribution (including waist circumference and skinfold thickness) showed that those parameters were better predictors for the development of asthma [17,18]. Although childhood obesity is indeed considered a global epidemic, few studies have assessed adiposity measures thoroughly, showing inconsistent results. Furthermore, epidemiological studies in children demonstrated that the risk of newly diagnosed asthma is not constant across the pediatric populations [19]. Several common disease-modifying factors including age, sex, ethnicity, the development of atopic conditions, and metabolic alterations significantly affect the onset and phenotypic characteristics of asthma [20,21]. The very point is if it could be possible to define different clinical subtypes of children with obesity and asthma.

**Figure 1:**
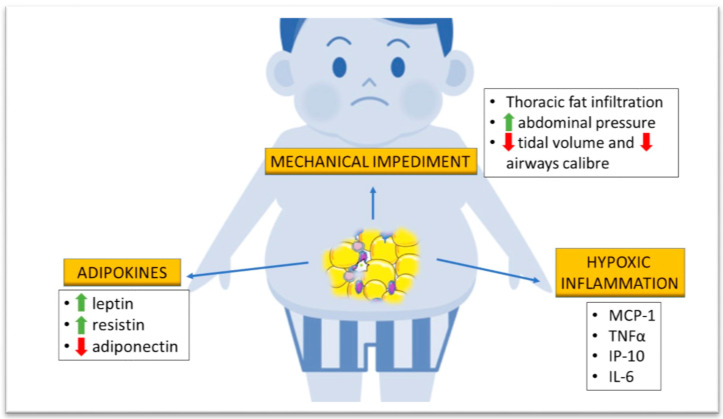
Pathological mechanisms of obesity that might influence lung function in children. Abdominal tissue deposition increases (upwards green arrow) abdominal pressure, forcing superficial ventilation and a consequent decrease (downwards red arrow) in tidal volume and airway caliber. Fat accumulation determines higher serum concentration of leptin and resistin, together with a decrease of circulating adiponectin. Excessive abdominal fat results in hypoxia with production of pro-inflammatory cytokines such as monocyte chemoattractant protein 1 (MCP-1), tumor necrosis factor α (TNFα), IFN-γ-inducible protein 10 (IP10), and interleukin-6 (IL-6).

The pathophysiological mechanisms at the basis of asthma and obesity correlation can be subdivided into three main categories: mechanical impediment, adipokine production, and hypoxic inflammation (Figure 1).

#### 3.2.1. Mechanical Impediment

Obesity reduces respiratory system compliance, lung volume, and airway caliber. The greater workload from adipose tissue deposition in the abdomen and chest wall of obese and overweight individuals forces them to breathe at lower tidal volumes, decreasing the stretching of airway smooth muscle (ASM) cells and increasing airway resistance over time [22]. Indeed, ASM stretching during air influx determines bronchodilation by means of actin–myosin bridge detachment [23]. Over time bronchial remodeling occurs, followed by airway hyperreactivity (AHR) and consequent reduction of lung volume, particularly the expiratory reserve volume (ERV) and functional residual capacity (FRC) [24].

#### 3.2.2. Adipokine Production

Adipose tissue represents an endocrine organ actively producing several different hormones that also regulate metabolism and inflammation. Excessive BMI is associated with greater circulating levels of leptin and resistin, together with a reduction of circulating adiponectin [25].

Leptin is produced in proportion to the adipocyte mass and acts as a proinflammatory hormone inducing neutrophil chemotaxis, active oxygen species production, natural killer cell and macrophage activation, and production of Th1 cytokines including interleukin(IL)-6 and interferon (IFN)-γ [26]. Interestingly, higher leptin levels seem to be associated with increasing risk of asthma development in children, particularly among atopic boys [27]. However, leptin does not modify eosinophilic inflammation and causes a shift from Th2 cytokine production to Th1 [27]. Rastogi et al. found an inverse correlation between leptin production and forced expiratory volume in 1 s (FEV_1_)/forced vital capacity (FVC) ratio in obese asthmatics [28]. This effect seems to be mediated by IFN-γ and IFN-γ-inducible protein 10 (IP-10), which are both Th1 inflammatory cytokines not associated with BMI. According to the authors’ hypothesis, leptin overproduction was responsible for the obstructive pattern in obese asthmatics by means of increased Th1 immunity [28].

Adiponectin appears to have a protective role against asthma. In animal models, it reduces allergen-induced AHR and suppresses eosinophil recruitment and Th2 cytokine expression in the lungs [29,30]. In humans, low adiponectin serum concentrations were found to be associated with a greater risk for asthma development, particularly among women and peri-pubertal girls [26]. In a cross-sectional study, pre-pubertal and peri-pubertal asthmatic boys with higher serum total adiponectin concentration showed less severe exercise-induced bronchoconstriction as well as fewer maximum asthma symptom days, fewer asthma exacerbations, higher FEV_1_/FVC ratio, and higher forced expiratory flow (FEF) 25–75% values [26].

There are limited scientific data on the relationship among serum resistin levels, BMI, and asthma. Kim et al. showed that in 149 atopic asthmatic children, 37 non-atopic asthmatic children, and 54 healthy age-matched controls, the serum resistin values were lower in atopic asthmatics compared with non-atopic asthmatics and healthy children [31]. Serum resistin demonstrated a positive correlation with the provocative concentration of methacholine causing a 20% drop in FEV_1_ (PC20) and a negative correlation with eosinophil count and serum total immunoglobulin (Ig)E as well. Ziora et al. determined serum levels of resistin in asthmatic children in relation to body weight, asthma severity, and gender, showing increased serum resistin values in children with atopic asthma [32]. Furthermore, both asthmatic-obese and normal-weight asthmatic girls as well as girls from the control group showed significantly higher mean serum resistin concentrations compared with boys from corresponding groups. These data would suggest an implication of this adipokine in the pathogenesis of asthma.

#### 3.2.3. Hypoxic Inflammation

Rapid and extensive adipose tissue deposition generates a hypoxic background that induces circulating monocytes to activate into patrolling monocytes, which produce inflammatory cytokines such as monocyte chemoattractant protein 1 (MCP-1), tumor necrosis factor (TNF), and IP-10, cytokines of the Th1 immune profile. The number of chemokine receptor type 2 (CCR2)-positive patrolling monocytes is inversely correlated with the FEV_1_/FVC ratio [33]. This polarization of the immune response might lead to neutrophilic bronchial infiltration, as demonstrated by Scott et al. in adult obese asthmatics [34]. Furthermore, obesity worsened the typical inflammatory background of asthma, increasing IL-6 production [34].

#### 3.2.4. Gut Microbiota

The gut microbiota is a complex microorganism population that plays an important role in host epithelial and immune functions. Its composition alterations are thought to be of paramount importance in the genesis of many diseases. Animal models showed that obesity is associated with peculiar modifications of the microbiota composition, with an increased ratio of *Firmicutes* to *Bacteroides* and a general reduction in biodiversity [35,36,37,38,39,40]. A recent study demonstrated that germ-free (GF) mice did not gain weight compared to conventionally-raised (CONV) mice, pointing out a pivotal role of gut microbiota in food energy intake [41]. Furthermore, GF mice showed greater weight gain after receiving fecal transplant from obese CONV mice compared with lean CONV mice [42]. Plasma, urine, and fecal metabolome analysis showed that obese subjects have greater concentration of small chain fatty acids (SCFAs) and biliary acids [43]. SCFAs regulate appetite, induce satiety hormones (principally glucose-like peptide 1, peptide YY, and leptin), increase energy expenditure, and have an anti-inflammatory effect by means of T regulator differentiation, inhibition of lipopolysaccharide-induced toll-like receptor activation, and production of TNFα [44]. Biliary acids have a bactericidal effect, attenuate inflammation, induce satiety, increase energy expenditure and glucose tolerance, and reduce lipid synthesis and gluconeogenesis [44]. Mice with low circulating SCFAs show greater allergic airway response and obese-asthmatic subjects have lower levels of bile acids (taurocholate and glycodeoxycholate) [45,46]. These findings might indicate a correlation between gut microbiota imbalances induced by obesity and asthma development. Unfortunately, current evidence is based mainly on animal studies and little is known about the effect of microbiota-derived metabolites on airway epithelium and smooth muscles in humans. It is likely that local cytokinic signals generated at the bowel endothelium level and metabolites produced by resident bacteria can lead to immune system activation and regulation at distal sites, but the exact molecular mechanisms linking gut microbiota alterations and lung function are still unknown [47]. A detailed analysis of asthmatic and obese patients’ metabolome will probably lead to new molecular pathways that would fill the present gap between the gut microbiota and these two major chronic diseases.

## 4. Common Obesity-Asthma Predisposition

Several studies support a common genetic predisposing background for asthma and obesity.

Hallstrand et al. analyzed the covariation between obesity and asthma in monozygotic and dizygotic same-sex twin pairs, concluding that approximately 8% of the genetic component of obesity is shared with asthma [48]. Polymorphisms of β3-adrenergic receptor, located primarily in adipose tissue, increase the capacity of gaining weight and are also associated with asthma phenotypes, severity, and β-agonist response. Moreover, TNFα gene haplotypes have been associated with asthma, AHR, and obesity [48]. A recent study analyzed the genetic variants associated with obesity and metabolic derangement in asthmatic individuals using a genome-wide association study (GWAS) [49]. The study population was divided into four asthma phenotypes: early-onset, late-onset, atopic, and non-atopic. Genetic obesity and glycemic traits were found to be positively associated with late-onset and non-atopic asthma, but not with early-onset and non-atopic asthma. No link was found between traits of dyslipidemia and any of the described asthma phenotypes. Interestingly, the major shared loci involved genes regulating inflammation and remodeling processes. For example, mothers against decapentaplegic homolog 3(*SMAD3*), which encodes a signaling transducer of transforming growth factor (TGF)-β, and erb-b2 receptor tyrosine kinase 3(*ERBB3*), a receptor of neuregulin implicated in inflammation regulation. In addition, both *SMAD3* and *ERBB3* control cell proliferation and differentiation. Other important shared genes were found to be implicated in extra-cellular matrix (ECM) deposition, such as Layilin(*LAYN*), encoding a hyaluronan receptor, and epithelial metaplasia, induced by forkhead box protein A3(*FOXA3)*, which determines goblet cell metaplasia. It is worth stating that causal inference analysis showed a unidirectional strong causal effect of BMI on asthma. This evidence is hampered by pleiotropy due to the polygenetic traits considered and by the cross-sectional design of the study.

However, genetic variants explain only a small proportion of asthma risk. Epidemiological studies established that fetal exposure to different risk factors alters gene expression and promotes the disease later in life. Maternal obesity and insulin resistance during pregnancy and the peri-conceptional period may influence lung development of the offspring. It can dampen glucocorticoid fetal production (indispensable for surfactant maturation), damage placental circulation by means of continuous background inflammation and oxidative stress, and expose the fetus to an over-nourished environment, inducing an obesogenic epigenetic footprint [50]. Therefore, maternal weight and metabolic control during pregnancy are of the uttermost importance to prevent obesity and asthma development in the offspring during childhood and later life, as recently stated by the World Health Organization [51].

Obese asthmatics show a different epigenetic footprint compared to obese non-asthmatic and normal-weight asthmatic children. It is characterized by hypomethylation of different promoters associated with innate immunity and non-atopic patterns of inflammation, including chemokine ligand type 5 (CCL5), interleukin 2 receptor antagonist (IL2RA), and T-box transcription factor 21 (TBX21), as well as hypermethylation of the high-affinity immunoglobulin E receptor (FCER) and transforming growth factor beta 1 (TGFB1) [52]. These epigenetic modifications might explain the Th1 polarization of immune responses observed in obese asthmatics, highlighting a completely different pathologic phenotype when both these conditions are present at the same time.

## 5. Sex Hormone Influence on Obesity–Asthma Link

The correlation between obesity, metabolic disease, and lung function is very complicated in the pediatric setting, as we have to consider a whole group of individuals with different age. This implies the need to assess every patient as a unique subject, given the continuous change of metabolic and physiologic backgrounds throughout child growth. Gender is another important factor that may influence metabolism, particularly during puberty. Adult epidemiologic studies found a greater prevalence of asthma in women than in men, probably mediated by circulating levels of estrogen [53,54]. The same prevalence pattern was outlined in a population of Brazilian adolescents aged 12–17 years [18]. A longitudinal study showed that the raise in BMI to overweight or obese range in girls during the pre-pubertal and peri-pubertal age was associated with increased risk of developing wheezing and asthma at 13 years of age; the same correlation was not observed in boys [55].

Estrogens can affect lung function by facilitating the release of IL-4 and IL-13 from peripheral blood mononuclear cells, two of the major Th2 profile cytokines involved in atopy and airway hyperreactivity [56]. In vitro studies showed that treatment of eosinophils with β-estradiol enhanced their adhesion to human mucosal micro-vascular endothelial cells and induced degranulation [57]. Even progesterone has the capability to increase bronchial eosinophilia and enhance bronchial responsiveness [58]. Furthermore, sexual hormone production induces a subcutaneous fat deposition pattern in peri-pubertal and post-pubertal girls, which represents the major source of leptin. This could explain why leptin determines AHR and asthma development particularly in females [34].

**Figure 2:**
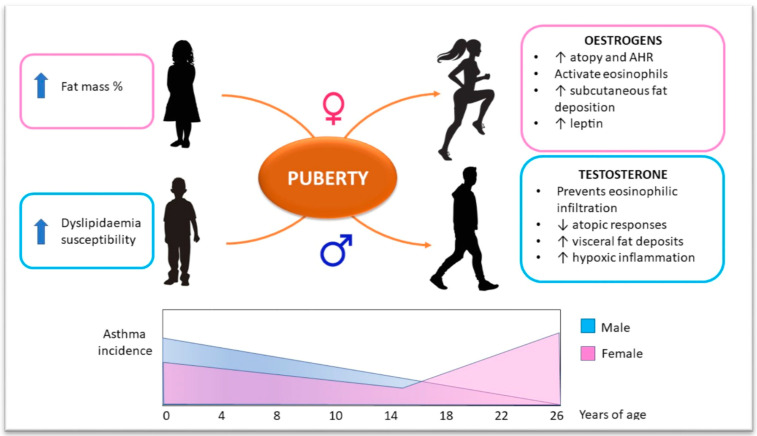
Sex hormones influence on the obesity–asthma link in children. Before puberty, asthma incidence is higher in males. Sex hormone production leads towards a shift in incidence ratio between genders. Estrogen effects are summarized in the right-hand pink box. Testosterone effects are summarized in the right-hand blue box. The diagram on the button schematically shows asthma incidence ratio in males and females from birth to early adulthood. AHR: airway hyperreactivity.

Gender difference in body weight gain and the risk of asthma development is present even in the pre-pubertal age, as demonstrated by Murray et al. [59]. In a prospective birth cohort study, they showed that the higher the BMI, the higher the risk of wheezing and eczema, especially among girls approaching middle childhood. Furthermore, there was no association between increasing BMI and atopy in boys or girls, suggesting mechanisms other than eosinophilic infiltration. Different proportions in body composition could explain the differences between males and females, with girls having a higher fat percentage than boys [59] (Figure 2).

Although there are many studies assessing the link between asthma and obesity in males and females, the debate on the role of gender in this relationship is still open. A longitudinal study in schoolchildren demonstrated a greater risk of asthma development among overweight boys compared with girls [60]. A recent metanalysis found no difference in asthma exacerbation rate and poor asthma control prevalence between obese boys and girls [61]. This discordance may be related to the wide variability in age range in different studies, with some authors taking into consideration only pre-pubertal children, and others considering adolescents only, or considering both groups. Pre-pubertal boys could develop dyslipidemia more frequently because of different diet habits (hypercaloric diet). Specifically, studies on the correlation between serum-circulating free fatty acids or low-density lipoproteins (LDLs) and asthma in obese children showed significant findings only among males [34,62]. More longitudinal studies are warranted to reliably estimate the difference in asthma development among obese and overweight boys and girls from infancy to early adulthood.

## 6. Different Clinical Subtypes of the Obese-Asthmatic Phenotype in Children

**Figure 3:**
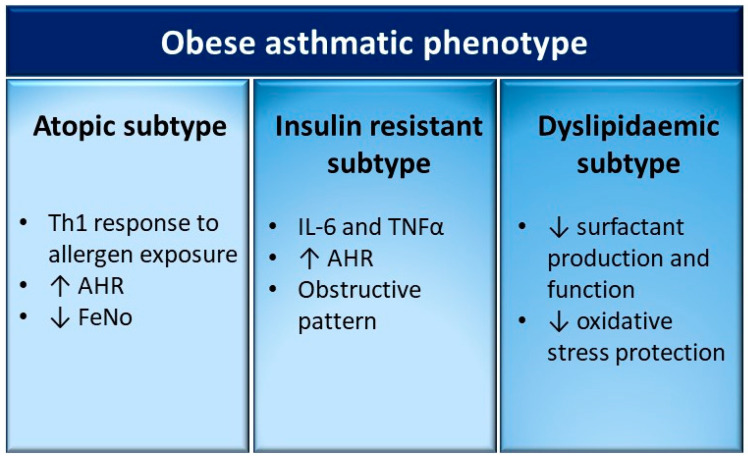
Potential clinical subtypes of the obese-asthmatic phenotype in children. The most relevant features of the three subtypes proposed are summarized in the relative columns. AHR: airway hyperreactivity; FeNO: fractional exhaled nitric oxide; IL-6: interleukin 6; TNFα: tumor necrosis factor α.

The graphic representation of the different clinical subtypes is shown in Figure 3.

### 6.1. Obese-Asthmatic Phenotype and Atopy

There are conflicting data on the relationship between atopy and obesity. Visness et al. found a dose–response relationship between BMI and sensitization to food-specific immunoglobulin (Ig)E, particularly in children aged 2 to 5 years [63]. Murine models showed that obesity induces AHR after allergen exposure through a non-Th2 response, as demonstrated by the absence of eosinophilic infiltration in the bronchial tree of challenged rats [64]. Similarly, challenging of serum from obese-asthmatic children and adolescents with dust mite allergens induces a Th1 immune response, characterized by higher circulating levels of MCP-1, TNF, and IP-10 [28,33]. In contrast, previous studies evaluating atopy by means of sensitization to inhalant allergens found no correlation with overweight or obesity [65,66]. Several groups have demonstrated a greater obesity effect on wheezing/asthma among non-atopic children. In a Boston area cohort, Taveras et al. showed in children from 1 to 3 years of age that high adiposity increased the odds of recurrent wheezing at age 3 by 46% [67]. However, obesity did not increase the risk for the atopic form of recurrent wheezing.

Several studies showed contrasting data on the association between BMI and fractional exhaled nitric oxide (FeNO), an indirect eosinophilic bronchial inflammation parameter. Some studies measured a lower FeNO concentration in obese asthmatics than normal weight asthmatics [68,69], while others found no difference between these two groups [70]. Consilvio et al. documented significantly higher FeNO values in asthmatic children compared to non-asthmatics, independently from BMI [71]. Furthermore, in a small number of studies, the BMI–FeNO relationship was significant only among non-asthmatic subjects [72,73].

There are clues in the literature of an interaction between atopy and dyslipidemia that favors the onset of asthma. In a cross-sectional study, asthmatic adolescents aged 16–18 years old had a twofold greater risk of having low circulating high-density lipoprotein (HDL) levels than their healthy peers. This correlation was more evident if the reduction of HDL levels took place during the peri-pubertal period and if there was a concomitant IgE sensitization [74]. Therefore, low HDL concentration, frequently found in obese subjects, might predispose to the onset of asthma when concomitant to the presence of atopy.

Given the heterogeneous evidence, it is quite complicated to exhaustively describe the link between atopy and obesity in asthmatic patients. It seems possible that chronic inflammation associated with obesity could worsen the atopic response of sensitized subjects in a Th2-independent manner. Further studies are requested to confirm this interesting hypothesis.

### 6.2. Obese-Asthmatic Phenotype and Insulin Resistance

Insulin shows a key role in regulating lung function and development. In a murine model, rats with induced type 1 diabetes mellitus, previously sensitized to ovalbumin, showed decreased atopic response after allergen exposure. Furthermore, the treatment of these rats with insulin injection recovered the atopic response, confirming that insulin plays a central role in allergy [75]. Studies on bovine tracheal smooth muscle (BTSM) demonstrated that insulin binds to specific receptors on ASM cell surfaces, directly inducing a pro-contractile status by means of laminin, calpontin, and myosin overproduction [76,77]. Furthermore, hyperinsulinemia inhibits pre-synaptic M2 muscarinic receptors, increasing acetylcholine release and subsequent ASM contraction [78].

Different human studies analyzed the prevalence of insulin resistance (IR) among the asthmatic pediatric population, finding a negative correlation with lung function independently from BMI and obesity [4,5,17,18,79]. A possible explanation could be that IR promotes the development of a Th1 inflammatory background through the production of many cytokines, such as IL-6 and TNFα [4]. The compensatory hyperinsulinemia induced by insulin resistance inhibits pre-synaptic M2 muscarinic receptors, increasing bronchial reactivity [80].

A cross-sectional survey study involving a nation-wide survey of the non-institutionalized population of the United States found that insulin resistance was associated with decreased FEV_1_ and FVC, whereas metabolic syndrome was associated with lower FEV_1_/FVC ratio [81]. These correlations were significant only among overweight or obese adolescents [81]. A recent cross-sectional study objectively measured the correlation among obesity, insulin resistance, and AHR using the mannitol challenge test in a group of pre-pubertal children [79]. The study findings showed a negative correlation between BMI and AHR, as well as between IR and AHR, but only the latter presented a linear pattern. In conclusion, the authors hypothesized that airway hyperreactivity in obese children is caused by hyperinsulinemia rather than obesity per se [79].

As already mentioned, insulin has a proinflammatory activity that induces a switch towards a Th1 immune response. The effect of insulin might therefore be similar to that of leptin, determining a reduction in FEV_1_/FVC ratio by a non-atopic mechanism. The association of obesity with insulin resistance is well established, but the latter might be present even in children with normal BMI. Sanchez et al. found that insulin resistance was associated with waist circumference and atopy, demonstrating that metabolic derangement could alter lung function even in normal weight children [17].

### 6.3. Obese-Asthmatic Phenotype and Dyslipidaemia

Obese subjects generally have high serum lipid concentration, particularly triglycerides and cholesterol, as well as a reduction of circulating HDLs. Certain epidemiological studies have evaluated serum lipid profile in obese asthmatics, obtaining interesting findings. Cottrell et al. demonstrated that high serum triglyceride values were correlated with chronic AHR independently from BMI [5]. Evaluating the concentration of circulating LDLs, Yang et al. showed that obese asthmatics presented the highest serum LDL and triglyceride concentrations, followed by non-obese asthmatics and then obese non-asthmatics [62]. The study finding highlighted that dyslipidemia was not only a parameter of obesity, but also an independent index of metabolic inflammatory derangement. It is worth noting that this relationship was significant only in males, probably due to different dietary habits between the two genders [62].

To date, the findings of the studies about the correlation between dyslipidemia and asthma are contradictory [82]. One potential explanation is represented by ethnic metabolic differences. A study on the National Health and Nutrition Examination Survey American population found a negative correlation of the total cholesterol and non-HDLs with current asthma only in the Mexican American group [83]. Several cellular and animal models demonstrated that LDL molecules are taken up by lung cells [84], where they suppress cellular responsiveness to TGF-β and inhibit histamine release from human lung mast cells and its effect [85,86,87]. This physiologic mechanism might explain the protective role of lipids on lung function at least in a selected subgroup of individuals. Therefore, ethnicity should be taken into consideration to better evaluate the relationship between dyslipidemia and asthma.

The metabolic pathways linking cholesterol and lung function are not fully understood. However, experimental studies have demonstrated that excessive amounts of total cholesterol impair surfactant synthesis, composition, and function, while HDL molecules serve as a major source of the antioxidant vitamin E for alveolar epithelial type II cells and promote surfactant production and lung fibroblast growth [74].

Non-alcoholic fatty liver disease (NAFLD) is a condition characterized by fat deposition into the liver and subsequent hepatocellular chronic inflammation that can progress in time to cirrhosis. NAFLD has become of great interest in recent years, emerging as the most common chronic liver disease in developed countries, not only in adults but also in children. Epidemiological data about the prevalence of NAFLD in childhood are variable, ranging from 7% to 38%, depending on the population examined [88]. A recent meta-analysis found an association between NAFLD and reduced predicted FEV_1_ and FVC in adults [89]. This relationship remained significant even after adjusting for age, sex, smoking history, BMI, waist circumference (WC), dyslipidemia, hypertension, and diabetes. It is worth noting that NAFLD severity seems to be directly proportional to abdominal obesity by inducing high abdominal pressure and increasing respiratory workload [90]. This could explain the correlation between decreased lung function and liver steatosis. Hong et al. found that NAFLD was linked with reduced lean body mass and respiratory muscle weakness, causing a restrictive pulmonary dysfunction at spirometry [91]. In addition, NAFLD is associated with insulin resistance and inflammation, whose effects on lung function have already been discussed [92,93]. In children only one cross-sectional study showed a strong correlation among NAFLD, BMI, signs of metabolic syndrome (such as insulin resistance and dyslipidemia), and asthma [88]. The overall prevalence of NAFLD was 15.8%, showing a rising trend from infancy to puberty. Interestingly, approximately 13% of asthmatic children in the study population showed fatty liver signs during an ultrasonography exam.

The relationship between dyslipidemia and asthma development is difficult to understand because there are multiple factors (dietary intake, ethnicity, sex, age) that could affect the pathological mechanism. Further studies are warranted to better understand the impact of NAFLD on asthma development in children and adolescents.

## 7. Conclusions

Several longitudinal observational studies hypothesized that childhood obesity may lead to asthma development. The asthma–obesity link in childhood is complicated and entangled, since it is affected by multiple modifying disease factors such as gender, adiposity measurements, atopy, insulin resistance, and dyslipidemia. Unfortunately, the heterogeneity of the evidence makes it difficult to define the impact of each single factor on asthma development in this population. The wide variability of results among several studies is mainly determined by different study populations and disparate tools adopted to assess asthma and obesity. Indeed, the majority of studies in children used questionnaires and medical history to diagnose asthma, choosing BMI to define obesity without measuring body fat distribution and lean mass percentage.

It is paramount to better understand the obese-asthmatic phenotype in children. Our revision of the literature allowed us to describe three main molecular mechanisms that might hypothetically underpin different clinical subtypes. The hypothesis to define different clinical subtypes might have an important impact for both future clinical management and research priorities. Firstly, it is known that obese asthmatic children with atopic sensitization show reduced Th2 inflammation and therefore a poor corticosteroid response. These patients might require alternative approaches besides inhalant steroid monotherapy. Secondly, the presence of IR in obese asthmatics may determine AHR and a more obstructive respiratory pattern, suggesting a potential better response to long acting bronchodilator therapy in addition to corticosteroid inhalants. Thirdly, obese asthmatic children with dyslipidemia might gain benefit from the use of cholesterol- and triglyceride-lowering drugs. Of course, to draw a different classification of the obese-asthma phenotype in children is beyond the scope of this review. In the end, our intention is only to hypothesize that the obese-asthmatic phenotype might be characterized by different clinical subtypes that would surely request a validation by large and well-conducted longitudinal studies. However, the current scientific evidence suggests that an early evaluation of metabolic risk profile among obese children may help to identify those at greater risk of developing pulmonary disease. This implies the necessity to study the obese asthmatic child with a “multidisciplinary approach”, evaluating the endocrinological and pneumological aspects simultaneously. This different approach would make it possible to intervene earlier in a specific manner, possibly with a personalized and tailored treatment.

## Figures and Tables

**Table 1:** Main cytokines involved in the asthma–obesity link [11]. Cytokines above the separation line are involved in Th2 response, while cytokines beneath the line are involved in Th1 response. Abbreviations: TSLP, thymic stromal lymphopoietin, IL, interleukin, MCP-1, monocyte chemoattractant protein 1, IP-10, interferon-γ inducible protein 10, TNF, tumor necrosis factor, IFN, interferon, TGF, transforming growth factor.

Cytokine	Source	Role
Alarmins (TSLP, IL-25, IL-33)	Epithelial cells	ILC2s and APCs activation after allergen exposure
IL-5	Activated ILC2s, mast-cells and Th2 lymphocytes	Promotes eosinophil proliferation and differentiation
IL-4	Th2 lymphocytes	IgE production, Th2 lymphocytes differentiation
IL-13	Mainly Th2 lymphocytes	promotes fibrosis, mucus secretion, IgE production, chemokines and adhesion molecules production
MCP-1 (CCL2)	Activated monocytes	Leukocytes migration to inflammation site
IP-10 (CXCL-11)	Activated monocytes	T-CD8+ migration to inflammation site
TNF	Activated monocytes, others	Neutrophils migration and activation, systemic inflammatory response
IL-1	Activated monocytes, others	Systemic inflammatory response
IFN-γ	NK cells, T-CD8+ cells, Th1 lymphocytes	Macrophages activation, Th1 differentiation, IgG production, MHC class I and II exposure
TGF-β	T lymphocytes, activated mononuclear monocytes	Inflammatory regulator, T-reg differentiation, IL-17 differentiation, IgA production, tissue repair
IL-17	Th17 lymphocytes	Neutrophils proliferation, differentiation and migration, induces IL-1, TNF and chemokines production
IL-6	Activated monocytes and T-lymphocytes	Promotes cellular-mediated response (↑ IL-17)
